# Racial Discrimination and Paranoia: Exploring the Potential Moderating Role of Ethnic Identity

**DOI:** 10.1192/bjo.2025.10118

**Published:** 2025-06-20

**Authors:** Rashmi Danwaththa Liyanage, Fiona Barlow, Pooja Saini, Taj Nathan, Jason McIntyre

**Affiliations:** ^1^Liverpool John Moores University, Liverpool, United Kingdom; ^2^Queensland University, Queensland, Australia; ^3^NHS, Cheshire and Wirral Partnership, United Kingdom

## Abstract

**Aims:** Psychosis is a severe mental health condition characterised by a loss of contact with reality, often manifesting in symptoms such as paranoid delusions and hallucinations. Research has shown that the prevalence of psychosis is significantly higher among individuals from ethnic minority backgrounds compared with White Britons in the UK. Additionally, people from these backgrounds tend to experience more discrimination and identity-related challenges than their White counterparts. However, few studies have explored how discrimination and ethnic identity intersect to influence psychosis rates.

This study aims to examine the relationship between discrimination and paranoia in ethnic minority populations, exploring whether ethnic identity may help protect individuals from the negative impact of discrimination on psychosis. It is hypothesised that people from ethnic minority backgrounds will face more discrimination and exhibit more severe symptoms of paranoia compared with White Britons, with the relationship between ethnicity and paranoia being mediated by discrimination. Furthermore, it is predicted that the association between discrimination and paranoia will be moderated by social identity, such that higher social identification will attenuate the positive association between discrimination and paranoia.

**Methods:** The study involved a secondary analysis of two cross-sectional datasets, including African and African Caribbean participants (N=355) and a student sample (N=1747), using validated measures of paranoia, psychosis symptoms (paranoia, dissociation, and hallucinations), discrimination, and ethnic identity.

**Results:** A moderated multiple regression indicated that the positive association between discrimination and paranoia was moderated by social identity, such that the effect was weaker and non-significant when people had stronger identities. We further found that discrimination mediated the effect of ethnicity (ethnic majority vs ethnic minority) on paranoia.

**Conclusion:** Strong social identities buffered ethnic minority people from the negative effects of discrimination on paranoia symptoms. Further, the higher rates of paranoia observed among ethnic minority participants may be explained by their more severe experiences of discrimination.

